# Gender Disparities Among Highly Cited Researchers in Biomedicine, 2014-2020

**DOI:** 10.1001/jamanetworkopen.2021.42513

**Published:** 2022-01-07

**Authors:** Amrollah Shamsi, Brady Lund, Mohammad Javad Mansourzadeh

**Affiliations:** 1 Independent researcher, Bushehr, Iran; 2 current PhD candidate, School of Library and Information Management, Emporia State University, Kansas City, Kansas; 3 Osteoporosis Research Center, Endocrinology and Metabolism Clinical Sciences Institute, Tehran University of Medical Sciences, Tehran, Iran

## Abstract

This cross-sectional study examines gender disparities in publication citations between men and women among highly cited researchers in the field of biomedicine.

## Introduction

Although the proportion of women authors in some medical fields has grown over the past decade,^1^ many authors suggest that articles authored by women tend to receive fewer citations than those authored by men.^2^ Receiving large amounts of citations can improve the odds of receiving grants and advancing one’s career.^2,3^ The inequities faced by women authors in some fields may produce significant hardship when trying to compete within academia. The purpose of this study was to examine the extent to which women are represented among highly cited researchers (HCRs) in the field of biomedicine.

## Methods

This cross-sectional study used data collected by Clarivate, which has created annual lists of HCRs from 2014 to 2020. We used these lists to identify the HCRs in 8 biomedicine fields (biology and biochemistry, clinical medicine, immunology, microbiology, molecular biology and genetics, pharmacology and toxicology, psychology and psychiatry, and neuroscience and behavior). To identify the gender of HCRs, we used the online gender-sorting tool Genderize.io (Demografix ApS), which has an accuracy threshold of at least 60%, similar to what has been done in prior studies.^1^ Authors with incomplete name data were excluded from the study. More information regarding our research methods can be found in the eMethods in the Supplement. Additionally, the 2-sided Pearson χ^2^ test was used to determine whether multiple affiliations were statistically significant between men and women HCRs. The level of significance was set at *P* ≤ .001. As secondary research using only publicly available data, this work was exempt from instutional review board review under the Common Rule (45 CFR §46). This study followed the Strengthening the Reporting of Observational Studies in Epidemiology (STROBE) reporting guideline.

## Results

Out of 10 915 HCRs, the gender of 10 656 (97.6%) was successfully identified. Of these 10 656 HCRs, 1710 (16.0%) were women and 8946 (84.0%) were men. The field with the largest percentage of women among highly cited authors was biology and biochemistry (21.5%), and the lowest was in clinical medicine (11.2%) (Figure, A). The linear equation of authors over the last 7 years showed a weak positive slope for the presence of women as HCRs; in 2014, 184 of 1343 HCRs were women (13.7%), compared with 293 of 1751 HCRs (16.7%) in 2020 (Figure, B). Compared with 2014, in 2020, women as HCRs had a declining trend in the fields of molecular biology and genetics (from 37 of 196 HCRs [18.9%] in 2014 to 25 of 203 [12.3%] in 2020; −6.6%) and microbiology (from 22 of 113 HCRs [19.5%] in 2014 to 17 of 128 [13.3%] in 2020; −6.2%), whereas the highest increasing trend was observed in neuroscience and behavior (from 12 of 129 HCRs [9.3%] in 2014 to 39 of 207 [18.8%] in 2020; +9.5%) and biology and biochemistry (from 29 of 193 HCRs [15.0%] in 2014 to 58 of 237 [24.5%] in 2020; +9.5%) (Figure, C). Overall, 2327 of 10 656 authors (21.8%) had multiple affiliations, of whom 278 (12.0%) were women (Table) (χ^2^_1_ = 37.082, *P* < .001). These findings suggest that women may be less likely to be affiliated with multiple institutions than men when authoring publications.

**Figure.**
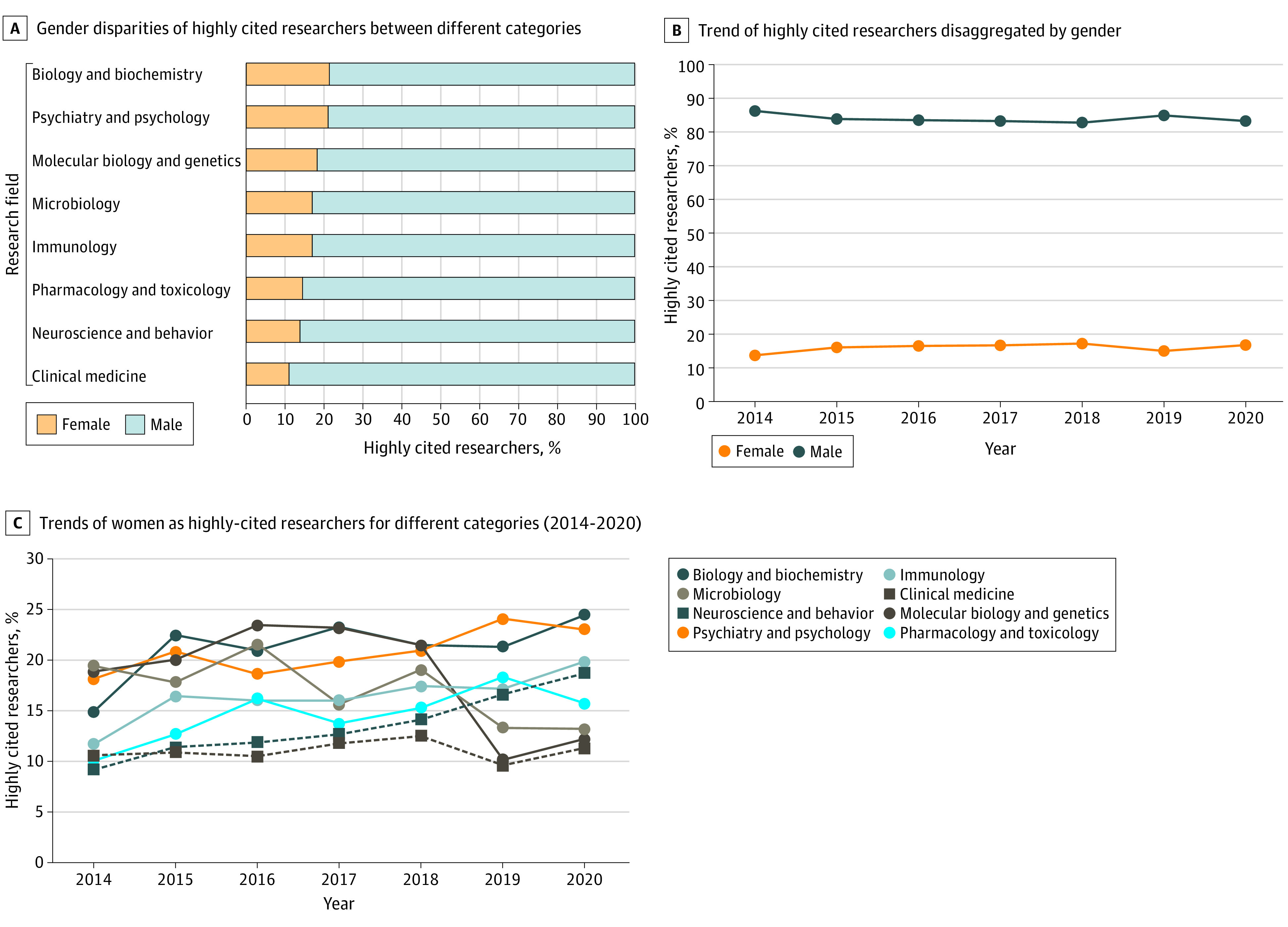
Gender Disparities Among Highly Cited Researchers in Different Research Areas and Over Time

**Table.** Comparisons of Single and Multiple Institutional Affiliations Among 10 653 Highly Cited Researchers in Biomedicine^a^

No. of affiliations	No. (%)			*P* value
	Total	Women	Men	
1	8326 (78.2)	1431 (83.7)	6895 (77.1)	<.001
≥2	2327 (21.8)	278 (16.3)	2049 (22.9)	

^a^ The affiliations of 3 authors were not provided in the Clarivate lists of highly cited researchers, so those 3 authors were excluded from this analysis.

## Discussion

Previous literature has shown that representation of women authors in high-impact biomedical journals is approximately 30%^1^; however, our study found that women represent only 16% of HCRs, providing evidence that women’s research in high-impact journals receives fewer citations than men.^2^ Although the percentage of women authors in biomedical journals is increasing,^1,2^ we found that articles authored by women receive considerably fewer citations on average. These findings suggest that considerable disparities remain in biomedical research authorship and popularity based on gender. According to our findings, women authors have the lowest representation in the clinical medicine category, which may raise concerns about women’s participation in macro-level, evidence-based health care decisions.^4^ Additionally, this study found that men hold more dual affiliations, which may mean that they have more resources for participation in high-impact research.^5^ Although gender disparities among researchers are not easy to overcome, increasing the number of women in prestigious biomedical research positions may boost both their research productivity and their likelihood of publishing highly cited publications.

Our study has some limitations. Some fields did not fit precisely or entirely within the category of biomedicine (such as psychology and psychiatry). However, there was no good way to divide these publications. Because the methodology for Clarivate’s listing of HCRs has changed since its inception in 2001, only recent HCR lists with the same methodology were used.

## Notes

Supplement. **eMethods.** Additional Methods
eReferences
