# Supplementary material for: Gender Disparities Among Highly Cited Researchers in Biomedicine, 2014-2020
Source: JAMA Netw Open. 2022 Jan 7;5(1):e2142513. doi: 10.1001/jamanetworkopen.2021.42513 (PMC8742200; doi:10.1001/jamanetworkopen.2021.42513)
Supplement: Supplement. — eMethods. Additional Methods eReferences [file jamanetwopen-e2142513-s001.pdf]

Supplemental Online Content

Shamsi A, Lund B, Mansourzadeh MJ. Gender disparities among highly cited researchers in biomedicine, 2014-2020. *JAMA Netw Open.* 2022;5(1):e2142513. doi:10.1001/jamanetworkopen.2021.42513

eMethods. Additional Methods

eReferences

This supplemental material has been provided by the authors to give readers additional information about their work.

© 2021 Shamsi A et al. *JAMA Network Open.*

eMethods. Additional Methods

The lists of Highly Cited Researchers™ (HCRs) for 2014 to 2020 were extracted from the Clarivate™[1]. The list of HCRs includes First Name, Middle Name, Family Name, ESI Category, Full Primary Affiliation, and Full Secondary Affiliation. HCRs are pioneer researchers who have demonstrated significant influence through publication of multiple papers, highly cited by their peers over the last decade. In the methodology of HCR, The Clarivate™ focuses only on highly cited papers[2] indexed in the Web of Science Core Collection™ during the last decade. HCRs are chosen for their outstanding achievements in one or more of 21 Essential Science Indicators (ESI) categories. The number of researchers selected in each category is based on the population of each field and calculated with the square root of the number of authors in each field. More details about the methodology of HCRs can be found in https://recognition.webofscience.com/awards/highly-cited/2020/methodology/. In this work we only selected fields that are related to biomedicine which were Biology and Biochemistry, Clinical Medicine, Immunology, Microbiology, Molecular Biology and Genetics, Pharmacology & Toxicology, Psychology and Psychiatry, and Neuroscience & Behavior.

To identify the gender of first names, we used Genderize.io algorithm. This database assigns genders to names based on the frequency with which they appear on public social media profiles where the user's gender may be verified. Genderize.io is widely used tool in for gender detection in academic studies (1-4). In this study we examined HCR names with Genderize.io. HCRs that their gender couldn't be identified with the probability ≥ 60% and the ones with incomplete names were excluded from the study.

We also analyzed gender disparities between single affiliation and multiple affiliations HCRs. To this aim we separate HCRs into two groups. First group was the HCRs with only one affiliation and the second group was the ones with ≥2 affiliations. Affiliation data is available in the lists of Highly Cited Researchers. The Pearson Chi-square test was used to determine whether multiple affiliations were statistically significant between men and women HCRs. The level of significance was set at a $P \leq 0.001$.

[1]- The archived lists of Highly Cited Researchers is available via: https://recognition.webofscience.com/awards/highly-cited/2020/

[2]- Highly Cited Papers are those that rank in the top 1% of citations in their field and year of publication.

© 2021 Shamsi A et al. *JAMA Network Open.*

eReferences

1- Hart KL, Perlis RH. Trends in proportion of women as authors of medical journal articles, 2008-2018. *JAMA internal medicine* 2019;179(9):1285-7. https://jamanetwork.com/journals/jamainternalmedicine/fullarticle/2733558

2- Thomas EG, Jayabalasingham B, Collins T, Geertzen J, Bui C, Dominici F. Gender disparities in invited commentary authorship in 2459 medical journals. *JAMA network open* 2019;2(10):e1913682-. https://jamanetwork.com/journals/jamanetworkopen/fullarticle/2753395

3- Chatterjee P, Werner RM. Gender disparity in citations in high-impact journal articles. *JAMA Network Open* 2021;4(7):e2114509-. https://jamanetwork.com/journals/jamanetworkopen/fullarticle/2781617

4- Volerman A, Arora VM, Cursio JF, Wei H, Press VG. Representation of women on National Institutes of Health study sections. *JAMA Network Open* 2021;4(2):e2037346-. https://jamanetwork.com/journals/jamanetworkopen/fullarticle/2776437

© 2021 Shamsi A et al. *JAMA Network Open.*
